# MANF Promotes Unexplained Recurrent Miscarriages by Interacting with NPM1 and Downregulating Trophoblast Cell Migration and Invasion

**DOI:** 10.7150/ijbs.85378

**Published:** 2024-01-01

**Authors:** Yuan Fang, Junhui Zhang, Damin Zhu, Qiong Mei, Ting Liao, Huiru Cheng, Ye He, Yunxia Cao, Zhaolian Wei

**Affiliations:** 1 Department of Obstetrics and Gynecology, the First Affiliated Hospital of Anhui Medical University, No. 218 Jixi Road, Hefei 230022, Anhui, China.; 2 Department of Obstetrics and Gynecology, The Second Affiliated Hospital of Anhui Medical University, Hefei 230022, Anhui, China.; 3 NHC Key Laboratory of Study on Abnormal Gametes and Reproductive Tract (Anhui Medical University), No. 81 Meishan Road, Hefei 230032, Anhui, China.; 4 Key Laboratory of Population Health Across Life Cycle (Anhui Medical University), Ministry of Education of the People's Republic of China, No. 81 Meishan Road, Hefei 230032, Anhui, China.; 5 Anhui Province Key Laboratory of Reproductive Health and Genetics, No. 81 Meishan Road, Hefei 230032, Anhui, China.; 6 Anhui Provincial Engineering Research Center of Biopreservation and Artificial Organs, No. 81 Meishan Road, Hefei 230032, Anhui, China.; 7 Anhui Provincial Institute of Translational Medicine, No. 81 Meishan Road, Hefei 230032, Anhui, China.; 8 School of Basic Medical Sciences, Anhui Medical University, Hefei 230022, Anhui, China.

**Keywords:** unexplained recurrent miscarriage, trophoblast cells, MANF, NPM1, p53

## Abstract

Dysplasia and invasive defects in early trophoblasts contribute to unexplained recurrent miscarriages (URMs). Mesencephalic astrocyte-derived neurotrophic factor (MANF) inhibits migration and invasion in some cancer cells, but its role in pregnancy-related diseases remains unresolved. Here, we found that MANF levels in the peripheral blood and aborted tissue of URM women were higher than in normal controls, irrespective of pregnancy or miscarriage. We confirm the interaction between MANF and nucleophosmin 1 (NPM1) in trophoblasts of URM patients, which increases the ubiquitination degradation of NPM1, leading to upregulation of the p53 signaling pathway and inhibition of cell proliferation, migration, and invasion ability. Using a URM mouse model, we found that MANF downregulation resulted in reduced fetal resorption; however, concomitant NPM1 downregulation led to increased abortion rates. These data indicate that MANF triggers miscarriage via NPM1 downregulation and p53 activation. Thus, MANF downregulation or disruption of the MANF-NPM1 interaction could be targets for URM therapeutics.

## Introduction

Recurrent miscarriage (RM) is defined as two or more spontaneous abortions that occur before the 20th week of pregnancy, with no emphasis on continuity. Most RMs occur in the first trimester of pregnancy in 1-3 % of couples trying to conceive 1-2. The causes of RMs are only identified in approximately 50 % of cases which include fetal genetic errors from chromosomal abnormalities in both parents, maternal endocrine factors, immune dysfunction, or anatomical abnormalities of the reproductive organs. The remaining cases are classified as unexplained RMs (URMs) and have recently gained more attention 3.

Successful implantation of the embryo is a key component of early pregnancy establishment. Post-implantation development and fetal growth depend on placental formation. As a component of the placenta, the chorionic trophoblast plays an important role in early pregnancy processes. Trophoblast cells originate from the trophoblastic ectoderm of the early embryo and gradually differentiate with development into villous cytotrophoblasts and extravillous trophoblasts (EVTs) 4. Most researchers believe that the trophoblast invades the endometrium and establishes a suitable microenvironment with the mother early in pregnancy, which is conducive to the implantation of the embryo. However, the healthy development of the placenta cannot be independent of the rational migration, invasion, proliferation, and apoptosis of trophoblastic cells, and most early URMs may be caused by the failure of these trophoblastic cell functions 5-7. Although trophoblastic cells are thought to be functionally involved in the pathogenesis of URM 8, little is known about the complex mechanisms regulating the migration and invasion of the trophoblastic layer at the maternal-fetal interface.

The matrix metalloproteinases (MMPs) family comprise proteases that promote the degradation of the extracellular matrix to break down its connectivity and regulate cell invasion into other tissues. The expression of MMP series proteins at different stages of pregnancy and pregnancy-related tissues is different 9. Gel proteases, MMP2 and MMP9, show the strongest localization on the placenta in early pregnancy and are mainly expressed in EVTs from six to eight weeks of pregnancy, regulating the role of early EVTs in invading decidua. By systematically detecting the mRNA and protein expression levels of MMPs throughout pregnancy, decidual stromal cells have higher MMP expression levels than that in trophoblasts, and the susceptibility of decidua to invasion is substantially increased in the presence of trophoblasts 10. In summary, to clarify the invasion of trophoblasts in URM during early pregnancy, we aimed to detect MMP2 and MMP9 at mRNA and protein levels in early miscarriage tissues (villi and decidua), respectively.

Mesencephalic astrocyte-derived neurotrophic factor (MANF) is an evolutionarily conserved neurotrophic factor, initially found to have a protective effect on midbrain dopamine neurons 11. MANF was later found to be expressed in a variety of peripheral non-neural tissues in mammals, including reproductive organs 12 and was determined to be primarily localized in the endoplasmic reticulum. MANF has been found to contribute to endoplasmic reticulum stress resistance in several neurological or metabolic diseases 13-14. The role of this neurotrophic factor in non-neuronal tissues depends on the tissue and cell type; MANF may be involved in the pathogenetic progression and prognosis of diseases such as hepatocellular carcinoma and colon and ovarian cancers 15-17 by inhibiting the migration and invasion of cancer cells (*in vitro*). This suggests that MANF has several cellular regulatory functions that include regulation of cell proliferation, apoptosis, and angiogenesis by countering endoplasmic reticulum stress or participating in inflammatory pathways 15.

The invasive ability of trophoblast cells in early embryos, although tightly regulated by the physiological environment, is similar to the development of cancer cells. However, the reduced invasive ability of trophoblast cells contributes directly to miscarriage. The role played by MANF in trophoblast cells is unknown. Here, we explored the expression levels of MANF in patients with URM and propose that the high levels observed may be involved in the pathogenesis of URM via interaction with the nucleolar phosphoprotein nucleophosmin 1 (NPM1), which results in decreased proliferation, invasion, and migration of trophoblast cells.

## Patients and Methods

### Clinical samples

We recruited patients who underwent an abortion in the Department of Gynecology of the First Affiliated Hospital of Anhui Medical University from December 2019 to December 2021 due to spontaneous abortion of early pregnancy and voluntary abandonment of pregnancy due to social factors. The inclusion criteria were as follows: the mother's age (20-40 years); two or more spontaneous abortions with the same sexual partner; intrauterine pregnancy confirmed by B-mode ultrasound; fetal cardiac arrest; and normal fetal karyotype as confirmed by high-throughput sequencing of the chorionic tissue after vacuum aspiration surgery. Exclusion criteria were as follows: genetic abnormalities in either parent; anatomical abnormalities of the reproductive organs in the mother; endocrine and metabolic diseases, autoimmune diseases; and infections. A total of 50 women with URM who met the above criteria and voluntarily participated in our study were defined as the URM group. As the control group, we recruited 30 healthy pregnant women who voluntarily terminated their pregnancy due to social factors (ultrasound indicated intrauterine pregnancy with normal fetal heartbeat) during the same period. General clinical information was collected from both groups, and venous blood and aborted tissue (villi and decidua) were collected on the day of the abortion procedure for subsequent analysis.

To determine whether pregnancy status and abortion affected our observations, we recruited 20 healthy female volunteers of childbearing age, defined as the non-pregnancy control group, at the physical examination center of the First Affiliated Hospital of Anhui Medical University where venous blood from these participants was collected. In addition, 19 patients in the URM group agreed to continue to participate in our study after the artificial abortion operation, and their venous blood was collected again for follow-up testing more than 6 months after the operation. This study was approved by the Ethics Committee of the First Affiliated Hospital of Anhui Medical University, and each participant signed a written informed consent form. The use of human samples conformed to the Declaration of Helsinki principles.

### ELISA assay

After blood collection from the participants and from experimental animals, the samples were processed for analysis. First, samples were placed at room temperature for 2 h or at 4 °C overnight, then centrifuged at 2500 rpm × *g* for 15 min. The supernatant was collected and preserved at - 80 °C until testing. Analysis of human (Abcam, Cambridge, MA, USA) and mouse MANF (CLOUD-CLONECORP, Wuhan, China) using ELISA kits was performed according to the manufacturer's instructions.

### Immunohistochemistry

Paraffin-embedded tissues were sectioned and heated at 60 °C for 1 h. Xylene-treated sections were dewaxed for 1 h and placed in gradient ethanol (100, 90, 80, 70, 60, and 50 %) for hydration. The sections were first treated with a solution containing 3 % Triton X-100 for 30 min, washed with phosphate-buffered saline (PBS, pH 7.2-7.4), and then placed in a 0.01 M citrate buffer (pH 6.0). The sections were then microwaved to 100 °C, maintained at this temperature for 20 min, and then cooled to room temperature. Immunohistochemical staining was performed according to the manufacturer's instructions (PV-9001 polink-2plus® polymer HRP kit; ZSGB-Bio, Beijing, China). The antibodies used were MANF (1:200; Abcam, Cambridge, MA, USA), matrix metallopeptidase (MMP)2 (1:400; Cell Signaling Technology, Danvers, MA, USA), MMP9 (1:400; Cell Signaling Technology), NPM1 (1:250; Proteintech, Chicago, USA), and proliferating cell nuclear antigen (PCNA, 1:200; Abcam). PCNA affects DNA replication and repair and is commonly considered a molecular marker of cell proliferation ability. Sections were imaged using an ortho-mounted fluorescence microscope (Olympus; Tokyo, Japan) and six representative fields were acquired per section. The index of positivity was quantified using the ImageJ software (NIH, Bethesda, MD, USA).

### TUNEL staining

Paraffin sections were routinely dewaxed and hydrated as described above. Apoptotic cells were labeled using DAB stain according to the manufacturer's instructions for the TUNEL Apoptosis Detection Kit (Abcam) and imaged using an ortho-fluorescence microscope. Similarly, the TUNEL Assay Kit with FITC Fluorescence was used to detect apoptosis in HTR-8/SVneo cells. Cells were uniformly inoculated in 6-well plates and fixed when confluence reached 60 %. According to the kit instructions, apoptotic cells will be labeled to show green fluorescence, and the results will be observed using laser confocal microscopy (Abcam).

### Western blotting

Total proteins were extracted from tissues and cells, using the RIPA buffer (Beyotime, Beijing, China) containing 2 % phosphatase inhibitors (Beyotime) and 1 % phenylmethanesulfonyl fluoride (PMSF; Beyotime). After denaturation, protein samples were resolved in sodium dodecyl sulfate-polyacrylamide gel electrophoresis (SDS-PAGE) and then transferred to polyvinylidene difluoride (PVDF) membranes (Sigma-Aldrich, St. Louis, MO, USA) which were blocked at room temperature for 2 h, using a 5 % skim milk buffer and then incubated overnight at 4 °C with different primary antibodies. The following day, depending on the species origin of the primary antibodies, the membranes were incubated with secondary antibodies bound to horseradish peroxidase at room temperature for 1 h. Protein blots were visualized using ECL chemiluminescent solution (Thermo Fisher Scientific, Waltham, MA, USA), and quantified using the Scion Image software (Scion Corporation, Frederick, MD, USA).

### RNA extraction and real-time PCR

Total RNA was extracted from villi and decidua tissues and cells using Trizol (Takara Bio, Shiga, Japan), and its concentration and purity were measured spectrophotometrically. RNA was reverse transcribed into cDNA using a reverse transcription kit (Takara). The SYBR Premix Ex Taq (Takara) was then used for real-time PCR assays. Primer synthesis was performed by Biotech Bio (Shanghai, China). Primer sequences are shown in Table S1 of the supplementary material.

### Cell culture, infection, and drug treatment

The human chorionic villous trophoblast cell line HTR-8/SVneo (purchased from the Cell Bank of the Chinese Academy of Sciences, Shanghai, China) was used for the in-vitro experiments. Cells were cultured in a DMEM/F12 medium (GIBCO, Billings, MT, USA) supplemented with 10 % fetal bovine serum (FBS; GIBCO), 1 % penicillin, and 1 % streptomycin (GIBCO) at 37 °C with 5 % CO_2_.

The packaged lentiviruses overexpressing MANF (oe-MANF), knocking down MANF (sh-MANF), and overexpressing NPM1 (oe-NPM1) were designed and synthesized by Gene-Chem (Shanghai, China). HTR-8/SVneo cells (6-well plates) were infected with oe-MANF lentivirus with a GFP fluorescent label, and the empty vector (oe-vector) was transduced in the control group cells. When more than 70 % of the cells were infected with lentivirus, purinomycin (2 μg/mL) was used to screen them and maintain stable MANF overexpression cell lines. Similarly, stable cell lines of sh-MANF and oe-NPM1 were produced using the same method. The small interfering RNA targeting NPM1 (si-NPM1) was used to transiently downregulate NPM1 expression. All stably expressed cell lines and the efficiency of concurrent transfections were evaluated using western blot analysis.

Nutlin-3 [a highly selective p53 protein agonist that inhibits the interaction between mouse double minute 2 (MDM2) and p53] and pifithrin-α (a classical p53 inhibitor) were used to modulate the endogenous levels of p53 in HTR-8/SVneo cells. Cells were then treated with 10 μM nutlin-3^18^ and 10 μM pifithrin-α 19, respectively, for 24 h. Control cells were treated with the same volume of DMSO.

### Cell proliferation assays (EdU and CCK-8)

Cells were cultured at 1 × 10^5^ cells per well in 6-well plates with glass coverslips, according to the manufacturer's instructions for the EdU kit (Thermo Fisher Scientific). When cells reached a confluence of approximately 60 %, the EdU working solution was added and incubated for 2 h at 37 °C. The cells were then fixed with 4 % paraformaldehyde and stained with Apollo 567 (30 min at room temperature) and 4′,6-diamidino-2-phenylindole (DAPI) for cell nuclear staining. Coverslips were imaged using a laser confocal microscope (Olympus) and analyzed with the ImageJ software.

For the cell counting kit-8 (CCK-8) assay (Beyotime Biotechnology, Shanghai, China), cells were cultured in 96-well plates. After 24 h, the cell culture medium was replaced with 10 μL of CCK-8 working solution and 90 μL of complete medium and cells were incubated for 3 h. The absorbance at 450 nm of optical density (OD) was measured using an enzyme marker (OD % = OD × 450 nm of the experimental condition).

### Wound-healing assay

To evaluate cell migration, we used the wound-healing assay. First, cells were inoculated in a 6-well plate and cultured to 100 % confluency to form fused monolayer cells. The cells were then scraped vertically, washed with PBS, and cultured for an additional 24 h in a culture medium containing 2 % FBS. The trace changes of scraped cells at 0 and 24 h were observed with an inverted fluorescence microscope (Olympus). Cell migration was assessed using Image J software analysis: degree of wound healing = 0 h scratch length (average of six field of view measurements) - 24 h scratch length (average of six field of view measurements) / 0 h scratch length × 100 %.

### Invasion assays

The invasiveness of cells was evaluated using the transwell assay (BD Biosciences, Bedford, MA, USA). The matrix glue (BD Biosciences) was diluted 6:1 in serum-free medium, evenly spread on the membrane in the upper layer of the transwell and placed in the incubator at 37 °C for 2 h to solidify. The cells were then diluted in a serum-free culture medium, added to the lower transwell chamber (200 μL of cell suspension; 8 × 10^4^ cells) along with 600 μL of culture medium with 10 % FBS, and incubated for 24 h. The cells were then fixed with 4 % paraformaldehyde (20 min), washed with PBS, and stained with a crystal violet solution for 15 min. Subsequently, the upper layer cells were air-dried and imaged in an inverted fluorescence microscope. The Image J software was used to analyze and quantify cell invasiveness, with cells stained with crystal violet defined as positive. The average number of stained cells in six random fields, in each compartment, was calculated.

### Immunoprecipitation (IP) / Co-immunoprecipitation (Co-IP)

Total proteins were extracted from the villi tissue using a NP40 lysis buffer (Beyotime) containing 1 % PMSF and 1 % cocktail (Sigma) on ice, with gentle movements. HA magnetic beads (Thermo Fisher Scientific) conjugated with the MANF primary antibody were incubated overnight at 4 °C. The following day, magnetic beads bound to MANF-associated proteins were washed, denatured, and analyzed using western blotting.

### Liquid chromatography with tandem mass spectrometry (LC-MS/MS) analysis of MANF-interacting proteins

LC-MS/MS was used to detect and screen for MANF protein interactors in the URM group villi. Co-IP experiments were carried out as described, followed by SDS-PAGE. Samples were visualized using a rapid silver staining kit (Beyotime). Protein band excisions were sent to Luming Biological Technology Co., Ltd. (Shanghai, China) for LC-MS/MS analysis.

### Glutathione S-transferase (GST) pull-down assay

We further determined the interaction between MANF and NPM1 using the GST pull-down assay. The plasmids of GST-NPM1 and HIS-MANF were transfected into *Escherichia coli*. *E. coli* cultures were collected, bound to HIS-labeled proteins with Ni2+resin for purification, and bound to GST-labeled proteins with GST resin. GST-labeled proteins and GST-NPM1 were incubated with GST beads at 4 °C for 4 h, respectively. After washing with PBS, the same amount of HIS-MANF was added and incubated at 4 °C overnight. After washing with PBS four times, 100 μL 2×loading buffer eluting beads was added, the contents were boiled for 10 min, and western blotting was used for analysis.

### CHX chase assay

CHX (100 μM) was added to HTR-8/SVneo cells overexpressing MANF and the vector control and treated for 0, 2, 4, and 8 h, respectively. Total proteins of cells at different treatment times were extracted for subsequent detection of NPM1 protein levels using western blotting, with GAPDH as the internal reference.

### *In vitro* ubiquitination assay

HTR-8/SVneo cells were treated in different groups, where each group of cells was treated with MG132 (20 μM, a potent proteasome inhibitor) for 6 h approximately 36 h after transfection. Each group of cells was collected for IP, as described above. Finally, the ubiquitination level of each group of cells was analyzed using western blotting.

### Analysis of the protein-protein interaction (PPI) network and KEGG pathway

Using the interaction database STRING—a platform for retrieving interacting genes or proteins; v.11.0—target genes that cross over with NPM1 were retrieved. The PPI network was constructed by entering NPM1 into the STRING online site and obtaining the top 10 genes closely related to it. KEGG enrichment analysis of target proteins closely related to NPM1 in the PPI network was performed using the clusterProfiler package in R (v.4.3.0), where a q value less than 0.05 was used as a screening condition for the dot plots.

### Establishment and treatment of the RM mouse model

As previously described 20, female CBA/J mice (6-10 weeks old) and male DBA/2J mice (8-10 weeks old) were purchased from Beijing Huafukang Biotechnology Co., Ltd. (Beijing, China). Male BALB/c mice (8-10 weeks old) in the control group were purchased from the experimental animal center of Anhui Medical University (Hefei, China). All mice were raised in standard conditions, with a constant temperature of 22-25 °C and 50-60 % humidity. All protocols were approved by the Experimental Animal Ethics Committee of Anhui Medical University (ethics number: LLSC20201138).

The animals were divided into two cohorts. The first consisted of three non-pregnant groups: the first group was a non-pregnant cohort where six CBA/J female mice (weighing > 20 g) were randomly selected and injected with 100 μL of knockdown MANF type 9 adeno-associated virus (AAV9-GFP-shMANF 21; synthesized by Gene-Chem) in the tail vein. The second group consisted of six additional CBA/J female mice, injected with 100 μL of the corresponding vector control, AAV9-GFP-vector. The third group corresponded to the blank control and consisted of six mice that did not undergo any treatment. Two weeks afterwards, the three groups of mice were euthanized, venous blood was collected, and the MANF serum concentration was measured using the mouse ELISA kit.

The second mouse cohort corresponded to the URM model. CBA/J female mice were mated with BALB/c male mice to establish a normal pregnancy model (n = 10) and with DBA/2J male mice for the URM group model (n = 15). The CBA/J female mice were then randomly selected for two treatments. The first group was injected with 100 μL AAV9-GFP-shMANF (n = 15; intravenous injection in the tail) and mated with DBA/2J male mice two weeks later. After mating, the vaginal plug was examined every morning and a positive vaginal embolism was defined as gestational day (GD)0.5. At GD14.5, females were euthanized, and the uterus, fetuses, and placenta were removed. The second group of females was similarly injected (100 μL AAV9-GFP-shMANF) and mated with DBA/2J male mice two weeks later. At GD0.5, 100 μL NSC348884 22 (5 mL/kg; n = 15; MedChemExpress, Monmouth Junction, NJ, USA) was injected intraperitoneally. Alternatively, 100 μL DMSO was administered to the control group (n = 6). The females were euthanized on GD14.5 and the uterus, fetuses, absorbed fetuses, placenta, and decidua were removed together and photographed for documentation. The decidua tissue was preserved for subsequent experiments. Fetal resorption rate = number of absorbed fetuses/total number of fetuses × 100 %. Supplementary Figure S1 shows a diagram of the animal model and the corresponding treatments.

### Statistical analysis

Statistical analysis was performed with SPSS (v.22.0; Chicago, IL, USA) and GraphPad Prism 8.0 (GraphPad Software, San Diego, CA, USA). Data are expressed as mean ± standard deviation. Comparisons between the two groups were assessed using an unpaired Student's t-test and between multiple groups using one-way ANOVA followed by Bonferroni correction (p < 0.05 was considered statistically significant).

## Results

### Clinical characteristics of the sample population

We compared the basic clinical information of the 30 women in the control group and the 50 women in the URM group. The participants did not differ statistically in age, body mass index, time since gestation, gravidity, or parity. The average number of pregnancy losses among women in the URM group was 1.55±2.67 (Table 1).

### Patients with URM had increased blood MANF levels and apoptosis in villi tissue and reduced levels of MMPs relative to those in the controls

**Figure 1:**
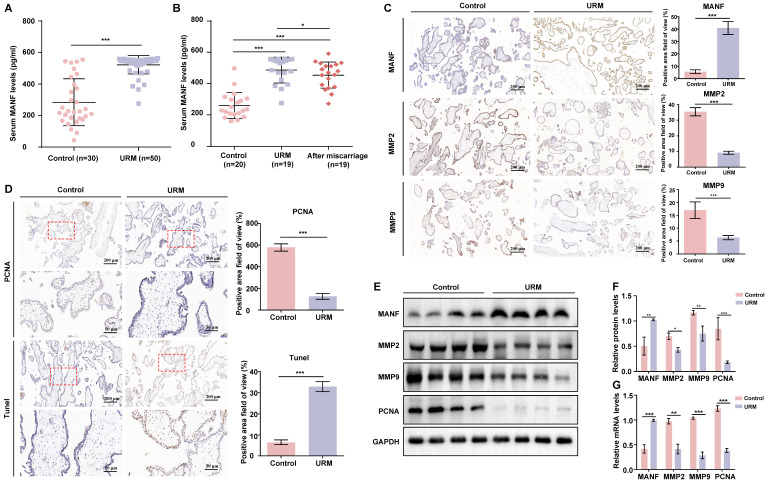
The levels of mesencephalic astrocyte-derived neurotrophic factor (MANF) in the serum and the expression levels of MANF, matrix metallopeptidase (MMP)2, and MMP9 in the villi of healthy pregnant women and pregnant women with unexplained recurrent miscarriage (URM), as well as cell proliferation and apoptosis. A. Comparison of MANF serum levels in 30 healthy pregnant women and 50 pregnant women with URM. B. Comparison of serum MANF levels in 20 healthy non-pregnant women of childbearing age and 19 women with URM, at the time of miscarriage and the non-pregnant period, more than 6 months after the abortion procedure. C. Immunohistochemistry of MANF, MMP2, and MMP9 in the villi of the two groups of participants, scale bar = 200 μm. D. Immunohistochemical staining of proliferating cell nuclear antigen (PCNA) and TUNEL staining of villi tissue, scale bar = 200 μm, 50 μm. E. Western blot analysis to detect the levels of MANF, MMP2, MMP9, and PCNA in villi from both groups. F. Immunoblot band density quantification. G. mRNA levels of MANF, MMP2, MMP9, and PCNA in the villi from the two groups. All results are from three or more independent experiments. * p < 0.05, ** p < 0.01, *** p < 0.001.

We first examined the MANF expression levels in the venous blood of women in the URM group (n = 50) which were significantly higher than those of healthy pregnant women (n = 30) (Figure 1A). To determine whether MANF expression levels correlated with gestational status or miscarriage, we also tested 20 healthy non-pregnant women and followed up on 19 women in the URM group 6 months after they underwent an artificial abortion. We observed that MANF expression levels were higher in the URM group compared to those in normal non-pregnant women, even after the patients with URM returned to the non-pregnancy status (Figure 1B). These observations were validated using immunohistochemistry, wherein we observed that MANF expression in the villi of the URM group increased significantly relative to the control group. Conversely, MMP2 and MMP9 expression decreased (Figure 1C). PCNA staining and the TUNEL assay were performed to detect cell proliferation levels and apoptosis in the villi tissues from both groups (Figure 1D). A significant increase in apoptosis and decrease in proliferation levels were observed in the trophoblast cells of participants in the URM group. The expression of MANF, MMP2, MMP9, and PCNA between the two groups of villi tissues was assessed by western blot analysis (Figure 1E and F). Consistent with the immunohistochemistry results, MANF levels increased in the villi tissues of the URM group participants while those in MMP2, MMP9 and PCNA decreased. A similar trend was observed for the mRNA levels (Figure 1G).

### The levels of MANF and apoptosis in the decidua of URM patients increase, while the levels of MMPs decrease

**Figure 2:**
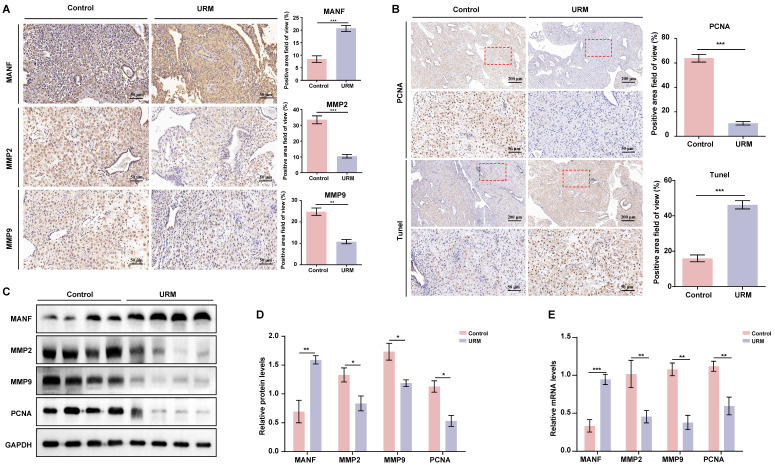
The expression level of MANF, MMP2, and MMP9 in the decidua of patients with URM, as well as cell proliferation and apoptosis. A. Immunohistochemical staining of MANF, MMP2, and MMP9 in the two groups of decidua, scale bar = 200 μm. B. Immunohistochemistry of PCNA and TUNEL staining of decidua tissue, scale bar = 200 μm, 50 μm. C. Western blot analysis to detect the levels of MANF, MMP2, MMP9, and PCNA in decidual tissue from both groups. D. Quantification of immunoblot band densities. E. mRNA levels of MANF, MMP2, MMP9, and PCNA in decidual tissue from the two groups. All results are from three or more independent experiments. * p < 0.05, ** p < 0.01, *** p < 0.001.

Next, we assessed the levels of MANF, MMP2, and MMP9 in decidual tissue from the two groups of participants, using immunohistochemical staining. In decidual tissue from the healthy participants, MANF levels were lower, whereas MMP2 and MMP9 levels were higher than those of the URM group (Figure 2A). PCNA and TUNEL staining showed that decidual stromal cells in the URM group had a significant decrease in proliferation and an increase in apoptosis (Figure 2B). Similarly, the protein expression analysis demonstrated that the results in the decidual tissue recapitulated those of the villi tissue. MANF expression in the decidua of the URM group increased, whereas the levels of MMP2 and MMP9 decreased (Figures 2C and 2D). The mRNA levels of these genes displayed similar trends (Figure 2E).

### Endogenous MANF levels affect the proliferation, migration, and invasion of trophoblasts

**Figure 3:**
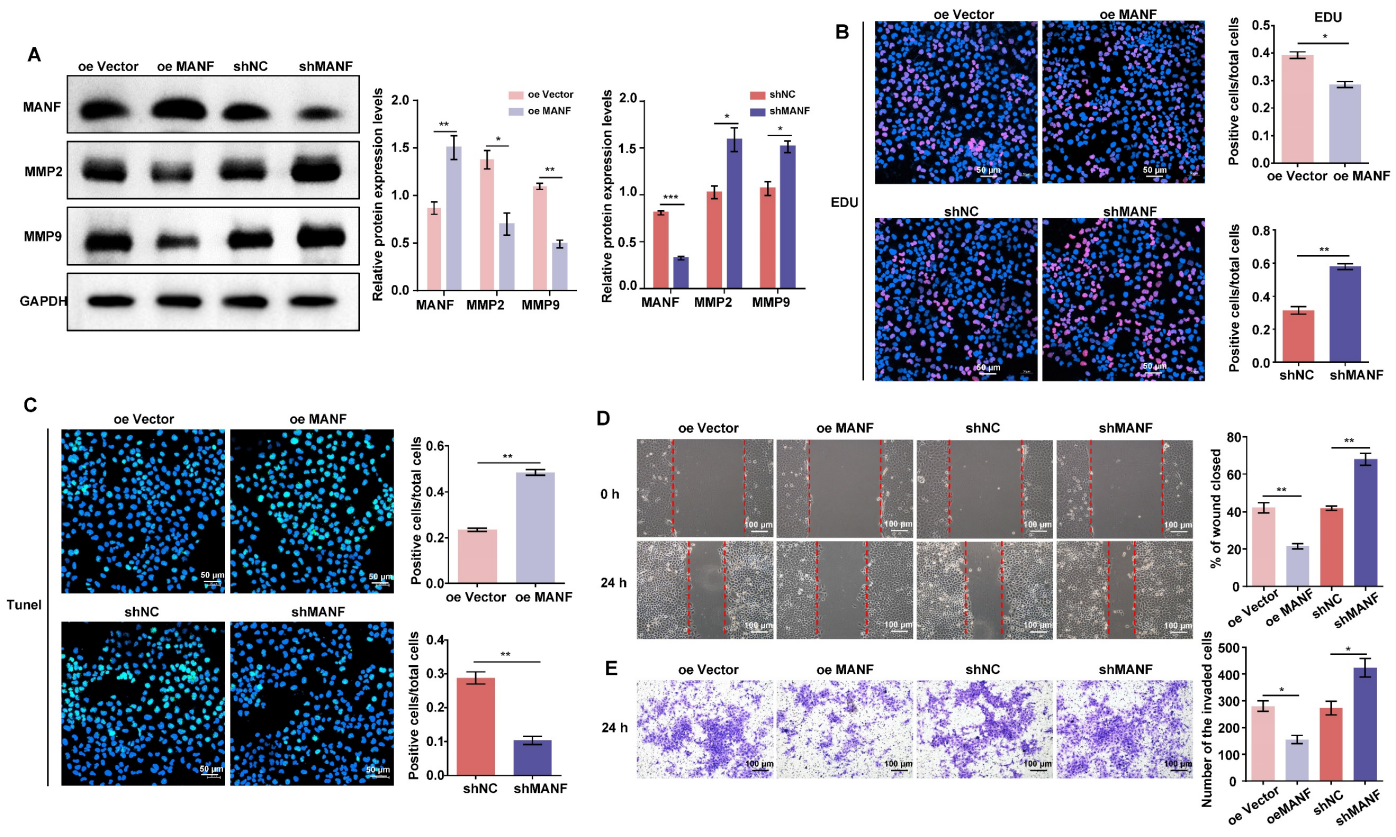
MANF expression levels differentially regulate MMP expression, as well as cell invasion and migration abilities. A. Levels of MMP2 and MMP9 in trophoblast cell lines with stable overexpression or knockdown of MANF, detected by western blotting. B. Proliferation of trophoblast cells at different levels of endogenous MANF assessed by EdU analysis, scale bar = 50 μm. C. Apoptosis of trophoblast cells at different levels of MANF using the TUNEL assay, scale bar = 50 μm. D. Wound healing assay showing the 24 h migration ability of trophoblasts expressing different MANF levels, scale bar = 100 μm. E. Transwell assay showing the 24-h invasion ability of trophoblast cells expressing different MANF levels, scale bar = 100 μm. All results are from three or more independent experiments. * p < 0.05, ** p < 0.01, *** p < 0.001.

As shown in Figure 3A, we produced stable HTR-8/SVneo cell lines overexpressing or knocked down for MANF (see Supplementary Materials Figure S2A and S2B for selection of stable cell lines with different knockdown MANF effects). We examined the expression of MMP2 and MMP9 in cells with different MANF levels. MMP2 and MMP9 expression decreased when the level of MANF increased; conversely, upon MANF knockdown, the levels of these MMPs increased. We also examined the cell proliferation and apoptosis levels in cells with MANF overexpression or knockdown using EdU and TUNEL staining. We observed that overexpression of MANF inhibited cell proliferation and promoted apoptosis (Figure 3B and 3C). Wound healing and transwell assays showed that overexpression of MANF was detrimental to cell migration and invasion, whereas MANF knockdown promoted both abilities (Figure 3D and 3E).

### MANF and NPM1 interact and reduce NPM1 expression in some trophoblast cells of patients with URM

**Figure 4:**
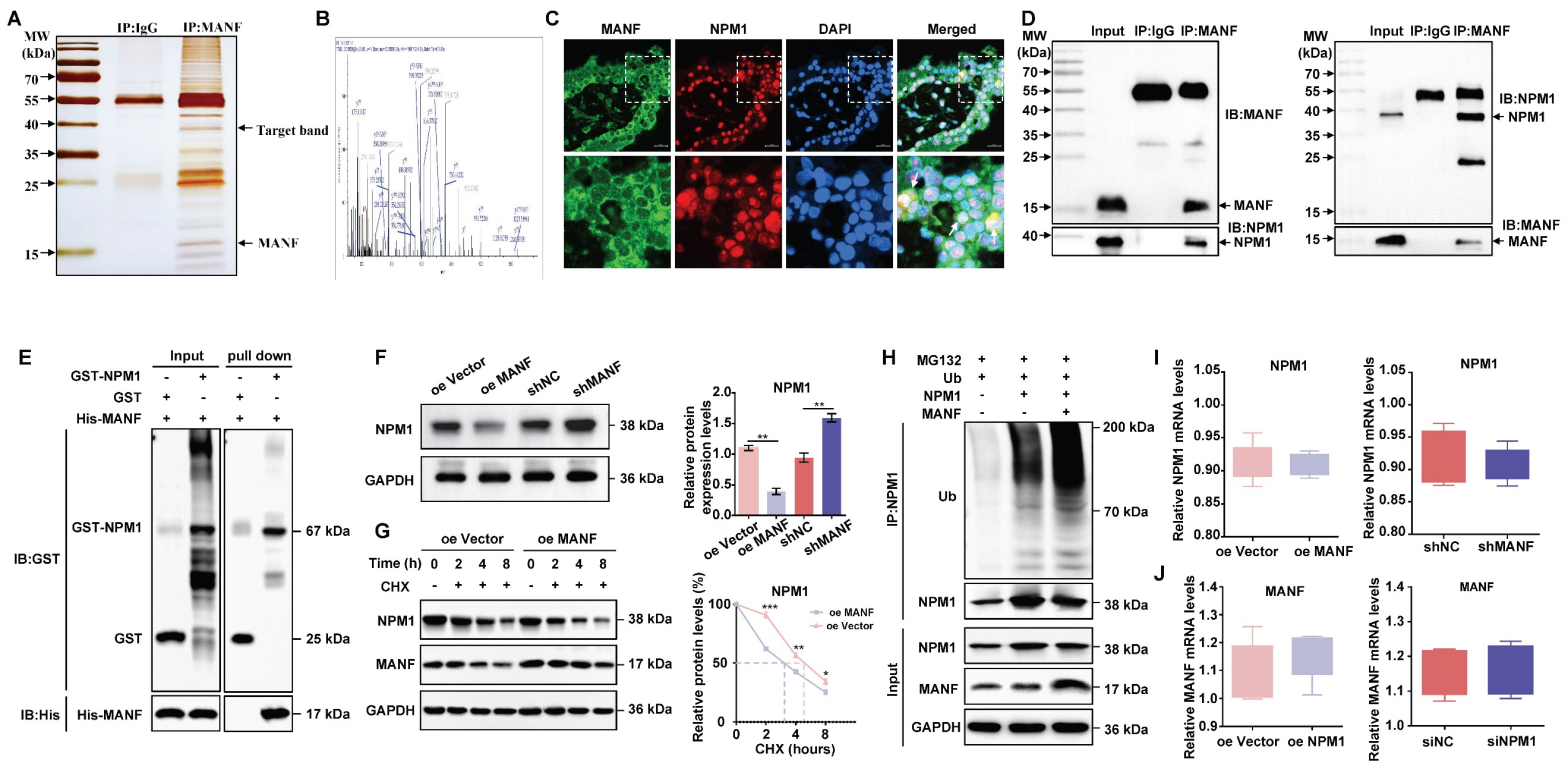
The interaction between MANF and NPM1 in the trophoblasts of URM patients leads to increased ubiquitination and degradation of NPM1. A. SDS-PAGE silver staining of co-IP samples using the MANF antibody in the villi tissue of patients in the URM group. MANF and target bands are labeled. B. Secondary mass spectra of NPM1-specific peptides identified by LC-MS/MS. C. Co-localization of MANF and NPM1 in the nucleus of trophoblast cells in the villi of patients with URM, detected by immunofluorescence double-labeling, scale bar = 100 μm. D. Co-IP confirming the interaction between MANF and NPM1 in the villi of patients with URM. E. A glutathione S-transferase (GST) pull-down assay revealed that MANF and NPM1 interact *in vitro*. F. Western blotting and statistical analysis of NPM1 levels in trophoblast cells with MANF overexpression or knockdown. G. Effect of addition of 100 μM CHX to HTR-8/SVneo cells overexpressing MANF and corresponding vector controls. Western blotting is used to detect changes in the half-life of the NPM1 protein and perform statistical analysis. H. Western blotting was used to detect the effect of adding or not adding MANF in HTR-8/SVneo cells on the ubiquitination level of NPM1. I. NPM1 mRNA levels in MANF overexpression or knockdown trophoblast cells. J. MANF mRNA levels in NPM1 overexpression or knockdown trophoblast cells. All results are from three or more independent experiments. * p < 0.05, ** p < 0.01, *** p < 0.001.

Next, we wanted to investigate potential MANF interactors. We performed co-IP on MANF and then visualized the protein interactors using rapid silver staining (Figure 4A). These interactors were then analyzed using LC-MS/MS. This analysis revealed that the nucleolar phosphoprotein NPM1, which is a protein that shuttles between the nucleus and cytoplasm and participates in functions, such as cell ribosome synthesis, centrosome replication, DNA repair, and cell apoptosis, is a potential MANF interactor (Figure 4B and Figure S3). Next, we evaluated the localization of MANF and NPM1 in the villi of URM patients using immunofluorescence dual labeling technology (Figure 4C). We observed that MANF had a nuclear translocation expression pattern in some trophoblastic cells (MANF is located in the endoplasmic reticulum of the cytoplasm under normal physiological conditions). MANF has nuclear colocalization with NPM1, and in these colocalization cells, we found that NPM1 lost its original nucleolar localization, with some being expressed in the cytoplasm. To further verify this point, we performed co-IP assays using antibodies against MANF and NPM1 (Figure 4D). The antibody against MANF immunoprecipitated NPM1 from the villi of patients with URM. Similarly, the NPM1 antibody was able to immunoprecipitate MANF, demonstrating the interaction between MANF and NPM1 in the villi of patients with URM. After using the GST pull-down assay to verify the direct interaction between MANF and NPM1 (Figure 4E), we confirmed that trophoblast cells with MANF overexpression showed a lower level of NPM1 than that in the controls, while inhibition of MANF expression upregulated NPM1 (Figure 4F). The CHX chase assay (Figure 4G) subsequently confirmed that overexpression of MANF proteins in trophoblast cells accelerated the protein half-life of NPM1. To further expand on these findings, we performed *in vitro* ubiquitination assays, and observed that MANF promoted ubiquitination degradation of NPM1 (Figure 4H), which we speculated may be the reason for decreased NPM1 protein levels after MANF overexpression. However, we found that changes in endogenous MANF levels in trophoblast cells did not affect NPM1 mRNA levels (Figure 4I). We constructed stable NPM1 overexpression and knockdown HTR-8/SVneo cell lines using lentivirus and siRNA (the selection of knockdown effects is shown in Figure S2C and S2D). Similarly, NPM1 did not affect the mRNA of MANF (Figure 4J).

### MANF may negatively regulate NPM1 in trophoblast cells, affecting cell invasion and migration ability

**Figure 5:**
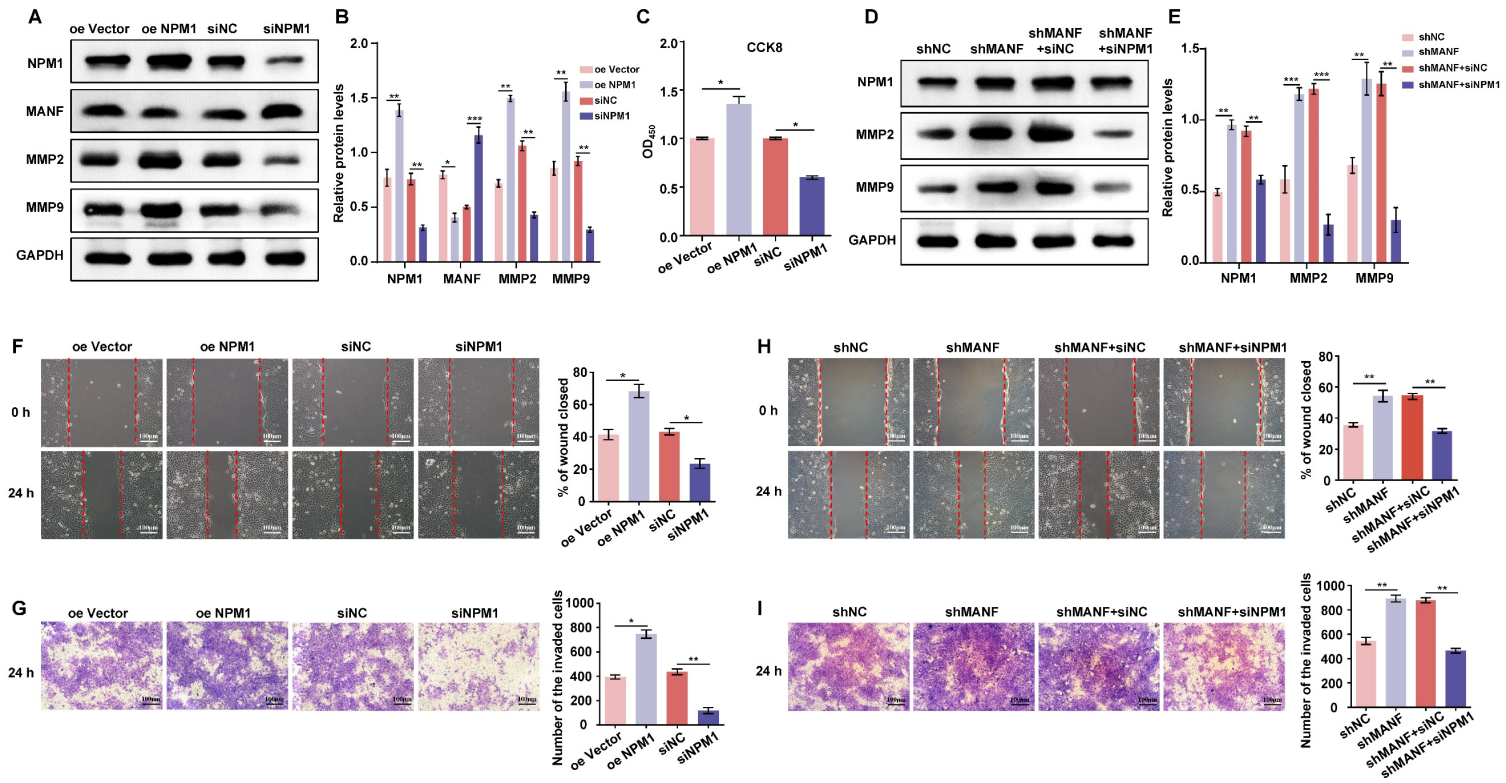
MANF negatively regulates NPM1 expression in trophoblast cells, affecting cell proliferation, invasion, and migration ability. A. Western blotting of MANF, MMP2, and MMP9 expression in trophoblast cells with NPM1 overexpression and knockdown. B. Western blot band density quantification. C. CCK-8assay showing the cell proliferation activity of trophoblast cells overexpressing and knocked down for NPM1. D. Western blotting of the expression of MMP2 and MMP9 after simultaneous knockdown of NPM1 and MANF in trophoblast cells. E. Immunoblot band density quantification. F. Results and statistical analysis of wound healing experiments in trophoblast cells with NPM1 overexpression and knockdown. G. Results and statistical analysis of 24-h transwell experiments in trophoblast cells with NPM1 overexpression and knockdown. H. Results and statistical analysis of wound healing in trophoblast cells with stable MANF knockdown and simultaneous inhibition of NPM1 for 24 h. I. Determination and statistical analysis of cell invasion capacity of trophoblast cells with stable MANF knockdown and simultaneous inhibition of NPM1 for 24 h. All results are from three or more independent experiments. * p < 0.05, ** p < 0.01, *** p < 0.001.

We examined the changes in the expression of MANF, MMP2, and MMP9 in trophoblast cells with knockdown and overexpression of NPM1 (Figure 5A and 5B). NPM1 expression was negatively correlated with the expression of MANF; however, NPM1 had a positive effect on the expression of MMP2 and MMP9. Indeed, high levels of NPM1 reduced the expression of MANF and enhanced MMP2 and MMP9 levels. Cell viability analysis using a CCK-8 kit showed that increased NPM1 levels promoted cell viability, whereas its knockdown decreased viability (Figure 5C). Next, we used transiently downregulated NPM1 (siNPM1) in a stable cell line with MANF knockdown to create a cell model with simultaneous low expression of MANF and NPM1 (Figure 5D and 5E). We compared this double knockdown with the cell line with MANF knockdown only. We found that the originally increased levels of NPM1 associated with MANF downregulation were reversed and that the increased levels of MMP2 and MMP9 were significantly downregulated by simultaneous disruption of NPM1 and MANF. We examined the impact of changing NPM1 expression levels on the ability of cells to invade and migrate using wound healing and transwell assays. We found that elevated levels of NPM1 promoted trophoblast invasion and migration whereas knockdown of NPM1 had the opposite effect (Figures 5F and 5G). Finally, using an siRNA of NPM1, we observed that the increase in cell migration and invasion capacity caused by the knockdown of MANF were partially reversed by inhibition of NPM1 (Figures 5H and 5I).

### Suppressed NPM1 expression in patients with URM leads to upregulation of p53 and inhibition of trophoblast proliferation, invasion, and migration

**Figure 6:**
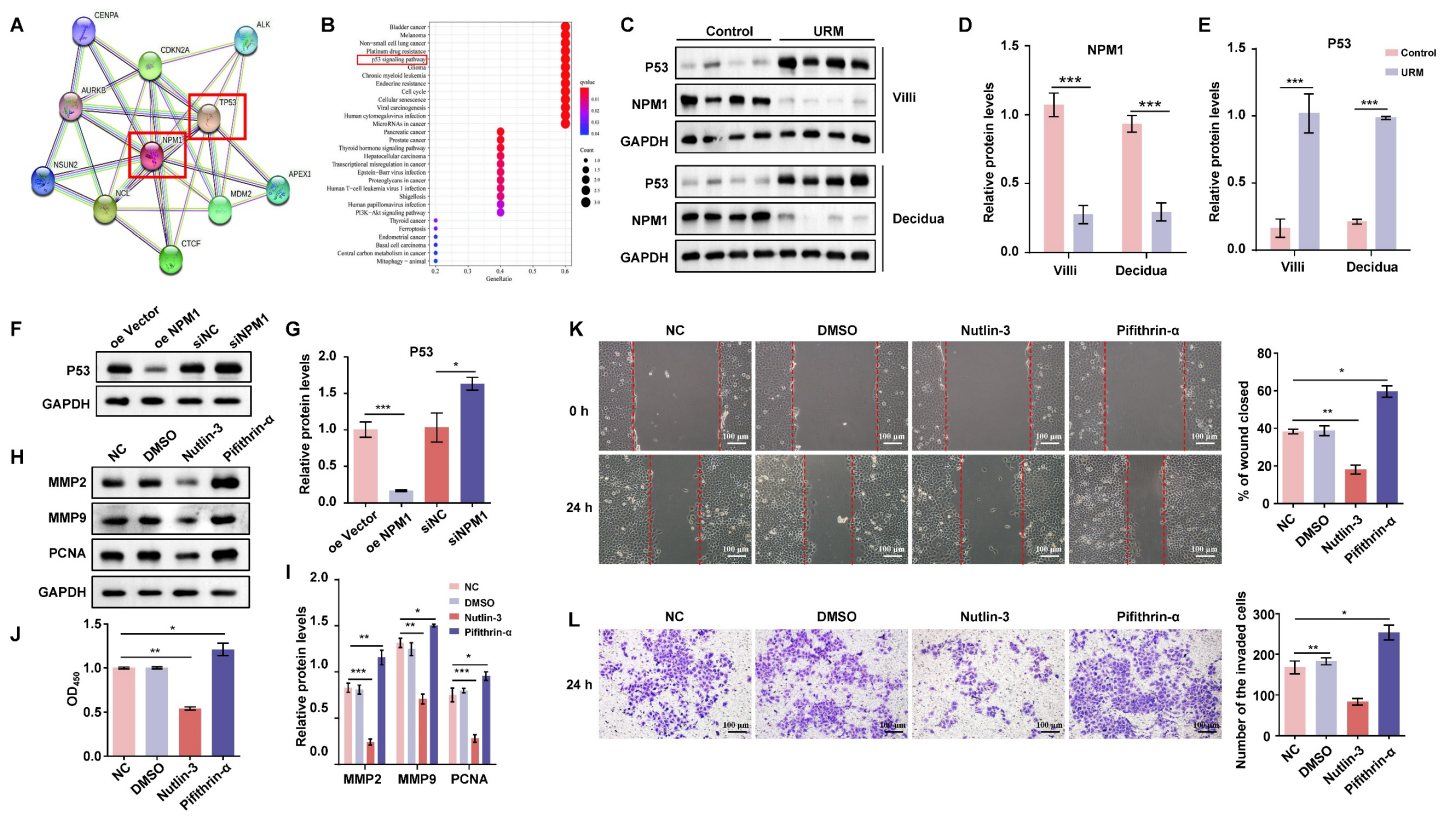
NPM1 is involved in trophoblast cell proliferation, migration, and invasion by regulating p53 protein expression. A. The PPI network was created using the top 10 genes with a close relationship to NPM1, retrieved from the STRING platform. B. Dot plots were produced after KEGG enrichment analysis of target proteins closely related to NPM1 in the PPI network. C, D and E. Western blot detection and statistical analysis of p53 and NPM1 protein expression in the villi and decidua from the control and URM groups. F and G. p53 protein levels and statistical analysis in trophoblast cells with NPM1 overexpression and knockdown. H and I. Western blotting and statistical analysis of MMP2, MMP9, and PCNA after treatment of trophoblast cells with p53 protein agonist nutlin-3 and inhibitor pifithrin-α. DMSO was used as a negative control. J. Cell viability assay using the CCK-8 kit after 24 h of nutlin-3 and pifithrin-α treatment in trophoblast cells. K. Results and statistical analysis of wound healing experiments with nutlin-3- and pifithrin-α-treated trophoblast cells. L. Transwell assays to detect the cell invasion ability in p53 drug-treated cells. All results are from three or more independent experiments. * p < 0.05, ** p < 0.01, *** p < 0.001.

Using the STRING database, we identified the 10 genes most closely associated with NPM1 and obtained a PPI network (Figure 6A). The P53 protein is highly associated with NPM1, and KEGG enrichment analysis of these 10 predicted genes revealed the involvement of the p53 signaling pathway (Figure 6B). To verify whether p53 is involved in URM pathogenesis, we first examined p53 and NPM1 expression changes in villi and decidua tissues in the control and URM groups by western blotting. We found that p53 was significantly upregulated in the URM group, while NPM1 levels decreased (Figure 6C, 6D, and 6E). In in-vitro experiments, as shown in Figure 6F and 6G, the effect of up- or downregulating NPM1 on p53 protein expression was analyzed. We observed that p53 expression was significantly increased when NPM1 expression was disturbed. Conversely, in trophoblast cells overexpressing NPM1, p53 expression displayed the opposite phenotype. The levels of PCNA, MMP2, and MMP9 in trophoblast cells were assessed using both the p53 agonist nutlin-3 and the inhibitor pifithrin-α. When p53 was activated, PCNA, MMP2, and MMP9 levels decreased (Figure 6H and 6I). The CCK-8 assay analysis confirmed that activation of p53 impairs cell proliferation and inhibition of p53 increases cell viability (Figure 6J). The wound healing assays and transwell analysis also showed a significant inhibitory effect of p53 protein activation on trophoblast cell invasion and migration (Figure 6K and Figure 6L). Treatment with the inhibitor pifithrin-α had the opposite effects.

### Reducing MANF levels in the URM mouse model reduces fetal resorption, but concomitant inhibition of NPM1 levels increases the probability of miscarriage

**Figure 7:**
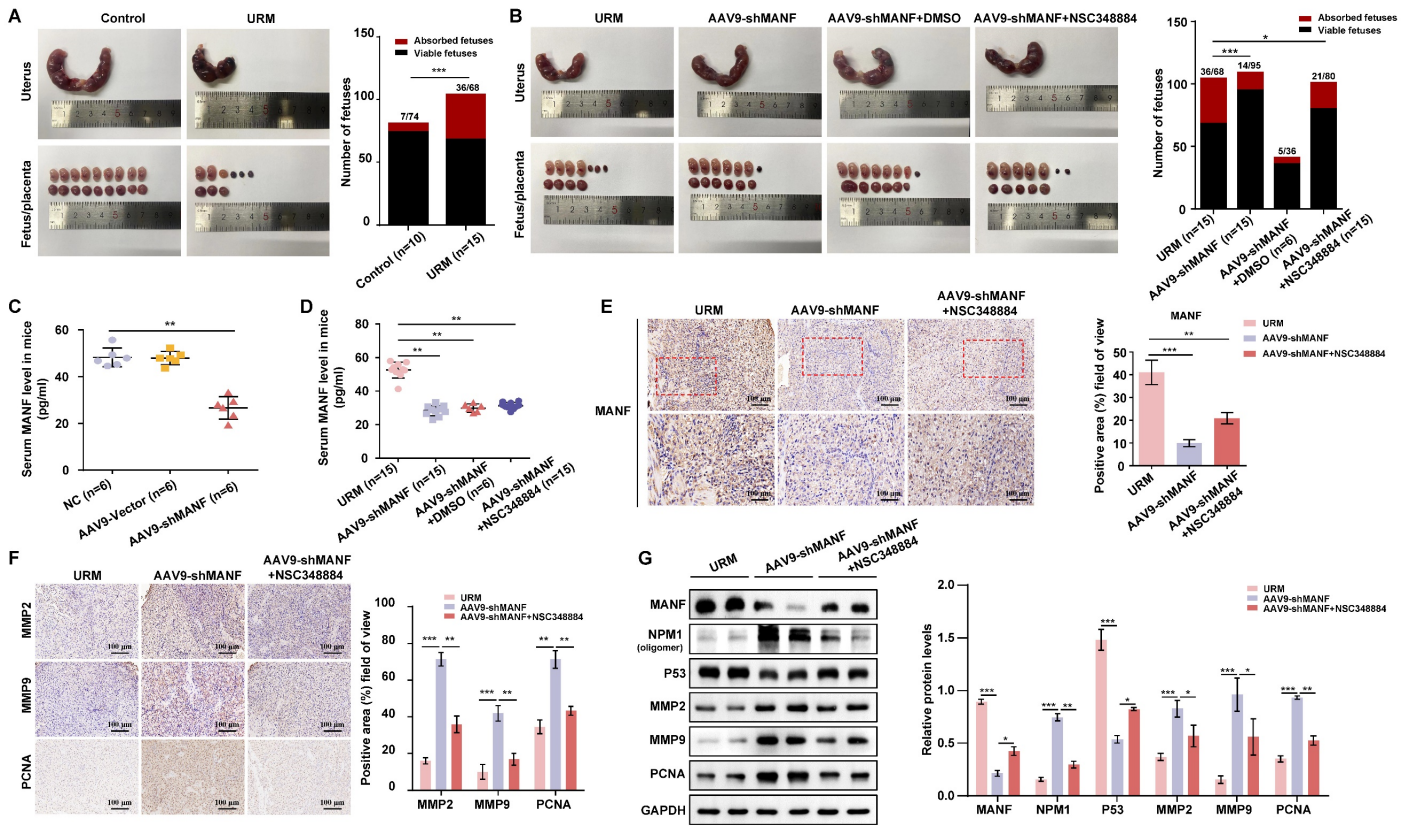
Establishment of the mouse URM model and the effect of MANF protein level changes on mouse pregnancy. A. Representative photos of the gestational day (GD)14.5 uterus, fetus, and placenta of the control (n = 10) and URM mice (n = 15), and statistics of absorbed fetuses versus surviving fetal mice. B. Comparative images of GD14.5 uterus, fetus, and placenta of the URM group (n = 15), AAV9-shMANF-treated group (n = 15), and AAV9-shMANF+NSC348884-treated group (n = 15), and statistics of absorbed fetuses versus surviving fetal mice. The AAV9-shMANF+DMSO condition (n = 6) was used as the NSC348884-treated vehicle control. C. MANF protein concentrations in the peripheral blood of CBA/J female mice after two weeks in the blank treatment group (n = 6), AAV9-shMANF (n = 6), and AAV9-vector (n = 6). D. MANF protein concentrations in peripheral blood in the URM group, AAV9-shMANF-treated group, and AAV9-shMANF+NSC348884-treated group at GD14.5, with the AAV9-shMANF+DMSO control. E. Representative images and statistical analysis of immunohistochemical staining of MANF protein in three groups of mouse decidua tissue, scale bar = 100 μm, 50 μm. F. Representative images and statistical analysis of immunohistochemical staining of MMP2, MMP9, and PCNA in mouse decidua tissue, scale bar = 100 μm. G. Levels of MANF, NPM1 oligomers, p53, MMP2, MMP9, and PCNA in decidua tissue detected by western blotting. All results are from three or more independent experiments, * p < 0.05, ** p < 0.01, *** p < 0.001.

The URM mouse model was used to validate our hypothesis on the role of increased MANF levels and the contribution to RM. We found that the incidence of fetal resorption in the URM group was significantly higher than that in the control group (p < 0.05; Figure 7A). Conversely, when we downregulated MANF expression in female mice (using AAV9-shMANF lentiviruses), we observed a reduction in the incidence of absorbed fetuses in the URM group. However, treatment with NSC348884 to interfere with the activity of NPM1 in mice with suppressed MANF levels, led to an increase in the probability of miscarriage, relative to the DMSO treatment control (Figure 7B). We used ELISA assays to analyze and compare the concentration of MANF in the peripheral blood of non-pregnant CBA/J female mice two weeks after injecting AAV9-shMANF in the tail vein, confirming the downregulation of this protein (Figure 7C). We also examined MANF concentrations in the serum of four groups of pregnant mice at GD14.5 and confirmed that the inhibitory effect of AAV9-shMANF remained stable during the gestation phase (Figure 7D). Considering that the absorbed fetal mice do not have a placenta, we selected the decidua tissue attached to the absorbed fetuses for subsequent testing. Specific MANF immunohistochemical staining was performed in these tissues of the URM, AAV9-shMANF-treated, and AAV9-shMANF+NSC348884-treated groups. We observed that MANF expression was significantly lower in the AAV9-shMANF group compared with that in the URM group, while MANF protein levels remained low after the inhibition of NPM1 (Figure 7E). We also assessed the levels of MMP2, MMP9, and PCNA in the decidua of these three animal groups (Figure 7F). We observed that MMP2 and MMP9 levels significantly increased in the AAV9-shMANF group; however, this effect was counteracted by inhibiting NPM1 with NSC348884. Similarly, the expression of PCNA in decidua tissues increased upon downregulation of MANF but inhibition of NPM1 activity impaired this effect. These observations are consistent with the results obtained using western blotting, where treatment with AAV9-shMANF reduced MANF levels in female mice, inhibited p53 protein activity by upregulating NPM1 expression, and ultimately promoted pregnancy progression by increasing PCNA, MMP2, and MMP9 levels, an effect reversed upon inhibition of the oligomeric activity of NPM1 (Figure 7G).

## Discussion

**Figure 8:**
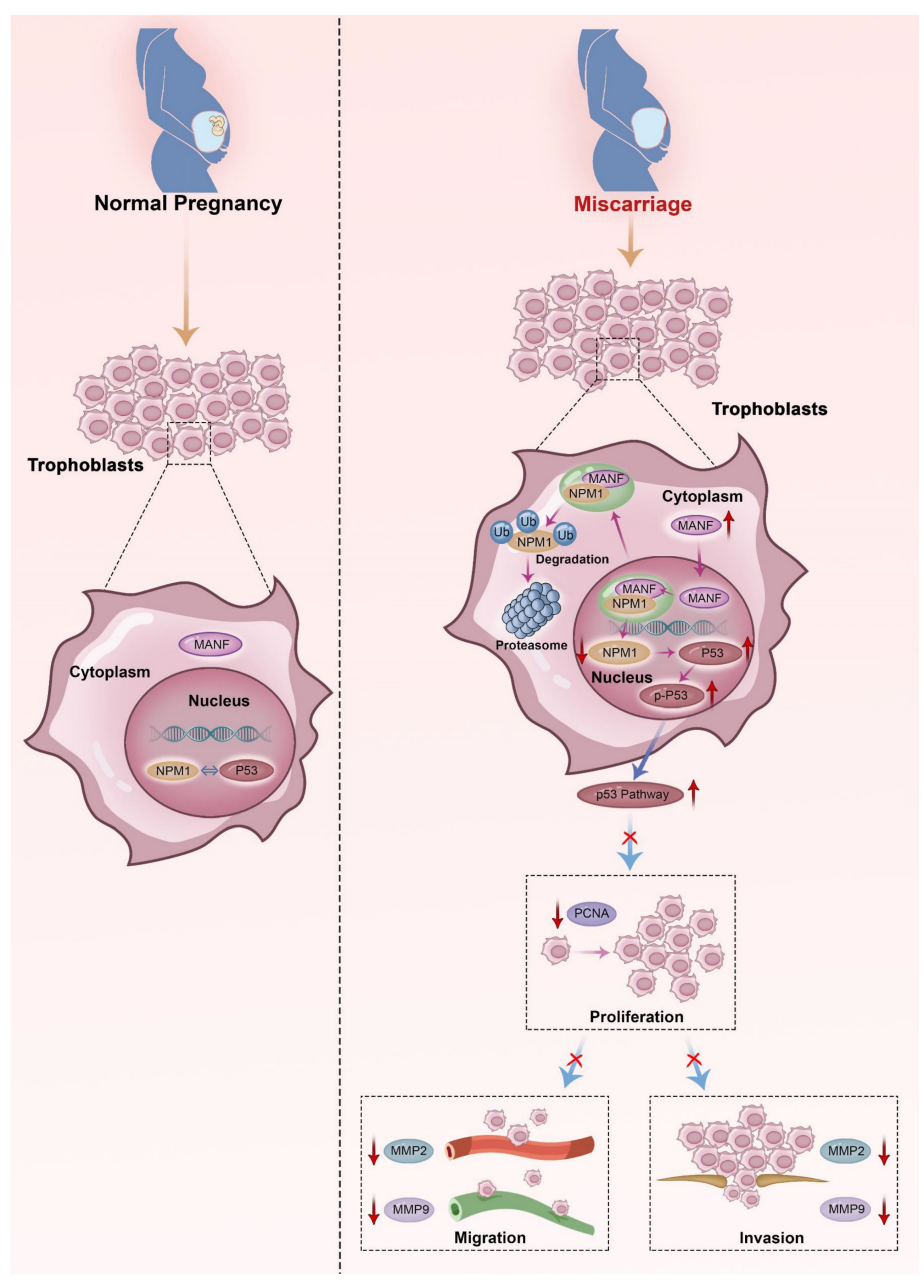
Schematic diagram of MANF and NPM1 interaction in the trophoblast during URM pathogenesis. In the trophoblasts of patients with URM, overexpressed MANF protein appears to undergo nuclear translocation and interacts with NPM1, resulting in increased ubiquitination of NPM1, decreased expression of NPM1, increased activation of the p53 signaling pathway, limited proliferation of trophoblastic cells, decreased invasion and migration functions, and induced miscarriage.

Here, we describe the role played by MANF, a non-traditional neurotrophic factor, in pregnancy-related diseases. Although reports of its expression and action in tissues outside the nervous system exist, to the best of our knowledge, no previous studies have linked MANF expression to RM. Here, we assessed the expression of MANF in patients with URM, which we found to be increased in the peripheral blood of these women compared to healthy pregnant women. These changes were found to not be due to pregnancy status or abortion. We propose that the abnormal elevation of MANF in trophoblasts of patients with URM disrupts the localization of NPM1 in the nucleus through interaction with NPM1, increases the ubiquitination level of NPM1, and leads to the degradation of the NPM1 protein, leading to an increase in p53 expression and upregulation of this signaling pathway. Activation of the p53 signaling pathway inhibits the proliferative function of trophoblast cells and reduces their invasive and migratory abilities, triggering the onset of miscarriage (Figure 8).

The most recognized MANF role is as a non-classical nerve growth factor that regulates neuronal cell survival and growth and acts as a protective agent to reduce neuronal cell damage during endoplasmic reticulum stress in neurons 23. MANF was initially named ARMET (arginine-rich mutated in early-stage tumors), with mutations found in a variety of solid tumors. However, studies linking MANF proteins and cancer have been scarce after mutations in this protein were found to be normal genetic polymorphisms 24. MANF was screened as the most sensitive gene to ER stress among 30,000 genes, using microarrays 25. This study also found that silencing of endogenous MANF expression with siRNA in HeLa cells—a cervical cancer cell line—increased cell proliferation ability, despite making the cells more susceptible to endoplasmic reticulum stress-induced death. These findings are consistent with our data showing that low levels of MANF induce trophoblast proliferation. MANF has been proposed as a potential therapeutic target for hepatocellular carcinoma 15. The authors suggested that MANF could suppress hepatocyte inflammation by inhibiting the NF-κB/Snail signaling pathway and reduce the invasive and migratory capacity of hepatocellular carcinoma cells by interfering with the hepatocyte epithelial-mesenchymal transition. Similarly, in a recent study on colorectal cancer, the authors found that DLC1 (deleted in liver cancer-1) controls cell migration and invasion ability by regulating the expression of secreted the MANF protein in colon adenocarcinoma cells and that cell invasion and migration ability decrease with increased MANF expression 16. These data support our experimental results that an elevated endogenous MANF protein concentration in trophoblast cells impairs cell migration and invasive functions.

NPM1 is a multifunctional protein that, under normal physiological conditions, localizes to the nucleolus and is involved in the assembly and transport of ribosomal proteins by shuttling between the nucleolus, nucleoplasm, and cytoplasm, regulating centrosome replication and participating in apoptosis and genome stability 26 Mutant NPM1 (namely, mutations located in exon 12) has been reported to be specific to acute myeloid leukemia 27. Mutant NPM1 promotes the migratory and invasive capacities of acute myeloid leukemia cells by upregulating MMP2 and MMP9 28. NPM1 has also been found to be associated with the progression of various human solid tumors, such as hepatocellular carcinoma, colon cancer, and prostate cancer, among other oncological diseases 29-31, and high levels of NPM1 can be observed in cancer tissue compared to paraneoplastic tissue. The ability of NPM1 to promote cancer cell migration and invasion has been verified *in vitro*. A previous report 32 examined the differential gene expression between the interimplantation and implantation sites in the mouse uterus on day 5 of pregnancy, using the SAGE (serial analysis of gene expression) technique. This study found that NPM1 is highly expressed at the implantation site. Additionally, Hu et al. 33 found that NPM1 may participate in embryo implantation by regulating nucleolar stress responses, and inhibition of NPM1 is a marker of nucleolar stress. Downregulation of NPM1 in early pregnancy impairs endometrial receptivity and reduces the implantation rate of pregnant mice. Accordingly, the physiological nucleolar stress responses may play a positive role in the decidualization process of mice and humans, which involves NPM1in the endometrium of pregnant mice. Inhibition of NPM1 expression interferes with normal nucleolar stress responses and mesenchymal-epithelial transformation, an important process of decidualization 34. NPM1 can occur in oligomeric, pentameric, and monomeric forms 35. The oligomeric state of NPM1 contributes to its nucleolar localization and is closely related to its role in cell proliferation; conversely, the monomeric form of NPM1 is associated with its participation in cellular DNA damage responses and induction of apoptosis 36. Therefore, the disruption of the oligomeric form of NPM1 affects its structure and function. In our animal studies, we selected NSC348884, a non-water-soluble compound that interacts with the highly hydrophobic NPM1 dimerization surface, which can target the NPM1 oligomeric form 37. This drug blocked NPM1 function by inhibiting the formation of NPM1 oligomers in mice, directly leading to an increased abortion rate. Recent studies on NPM1 and endometrial cancer have shown that the expression level of NPM1 has a positive regulatory effect on the proliferation, migration, and invasion of endometrial cancer cells 38, consistent with the results obtained in our in-vitro experiments. Trophoblasts have a highly similar invasive ability to cancer cells. When siRNA was used to downregulate NPM1 in HTR-8/SVneo cells, the migration and invasion rate of these cells was significantly reduced.

The protein p53 (also known as TP53) was discovered in 1979 39 and is a key tumor suppressor. This protein is a negative regulator of the cell cycle in physiological conditions and is related to other important biological functions such as DNA repair, cell differentiation, and apoptosis. When external environmental stresses lead to cellular DNA disorders, p53 is activated to stop cell cycle progression and mediate apoptosis 40. The level and activity of p53 are strictly controlled by the organism under physiological conditions, with the MDM2 oncogene as the main p53 cellular regulator. MDM2 promotes p53 protein degradation and inhibits p53-mediated gene expression by forming a complex with this protein. Mice lacking MDM2 do not survive; conversely, those lacking both MDM2 and p53 develop normally and survive, suggesting that MDM2 functions *in vivo* via reciprocal regulation with p53. Additionally, the transcriptional activation of p53 is inhibited through interaction with MDM2 41-42. The p53 protein agonist nutlin-3 and the inhibitor pifithrin-α, used here, regulate p53 protein levels by targeting the formation of the MDM2-p53 complex. Both drugs have been widely used in in-vivo and in-vitro experiments for p53 research 43. NPM1 protein overexpression and uncontrolled expression are associated with accelerated cell growth. This nucleolar protein has been proposed to directly interact with p53 to regulate its stability and increase the transcriptional activity of p53 following different types of stress 44. However, this proposed NPM1 function remains controversial, with some reports of this interaction occurring in cells that are in hypoxic environments, which inhibits hypoxia-induced activation of p53 (via phosphorylation at Ser15), thereby attenuating apoptosis induction 45. Maiguel et al. 46 reported that NPM1 is an early responder to DNA damage in cells, using a DNA damage model induced by ultraviolet radiation, which can prevent premature activation of p53. By inhibiting p53 activation (i.e., phosphorylation at Ser15), these researchers were able to inhibit p53-dependent apoptosis. Overexpression of NPM1 in cancer cells may lead to p53 protein inactivation and accelerate cancer progression. In a mouse model, in cells lacking NPM1, a DNA damage response is activated, promoting p53 activation and leading to cellular senescence or apoptosis 47. Additionally, NPM1 knockdown in neural stem cells led to a significant increase in p53 levels and its downstream target gene p21; these cells underwent significant apoptosis via the p53 signaling pathway 48. This evidence suggests that there is a mutual regulatory mechanism between NPM1 and p53. Here, we found that the knockdown of endogenous NPM1 leads to p53 upregulation in trophoblast cells, affecting cell migratory and invasive properties. These observations are in accordance with the findings from most studies, already discussed.

The role of the p53 signaling pathway in miscarriage is well established. The elevated levels of p53 in cytotrophoblast cells, as well as the elevated expression of its downstream apoptosis-inducing p21 and Bak proteins, along with decreased levels of MDM2, lead to the activation of apoptosis in these cells 49. Wang et al. also detected abnormally high levels of the p53 protein in the villi tissue of patients with RM, which is involved in the mechanism of miscarriage by regulating the proliferation, migration, and invasive capacities of the human trophoblast cell line, HTR-8/SVneo 50. Overexpression of the p53 protein in abortion-associated tissues of URM patients in our study may be related to the downregulation of NPM1 by MANF, resulting in increased apoptosis and reduced migration and invasion of trophoblast cells, as confirmed in our URM animal model.

Here, MANF was found to be expressed at high levels in patients with URM and this differential expression was not affected by pregnancy or miscarriage. We observed that MANF co-localized and interacted with NPM1 in the nucleus of trophoblast cells from URM samples, interfering with NPM1 expression, a mechanism that may be involved in the process of miscarriage. However, this study had some limitations: a larger sample size is needed to validate MANF expression changes in patients; MANF translocation from the cytoplasm to the nucleus was observed in some trophoblast cells from patients, which could be due to outside stress or genetic alterations. The cause of this subcellular localization change needs to be elucidated to shed light on the molecular mechanisms underlying URM.

## Conclusions

This study revealed a potential role for MANF in the URM population, a finding that enriches the literature on the function of this neurotrophic factor in non-neuronal cells. Overexpression of MANF was found to limit the proliferation of trophoblast cells and their migratory and invasive functions. Conversely, MANF downregulation in mice was shown to reverse these effects on the occurrence of miscarriage in animal experiments. The specific causes of elevated MANF and nuclear ectopia in patients with URM need to be further explored to elucidate the potential therapeutic interventions of URM.

## Supplementary Material

Supplementary figures and table.

## Figures and Tables

**Table 1:** Basic clinical characteristics of the included patients.

Group	Control	URM	p-value
n	30	50	
Age (years)	27.56 ± 4.39	29.14 ± 2.76	0.413
BMI (kg/m^2^)	22.01 ± 2.17	21.49 ± 3.71	0.577
Pregnant (times)	2.67±0.66	2.76±0.82	0.149
Parity (times)	0.33±0.47	0.22±0.42	0.463
Pregnancy loss (times)	0	2.54±0.68	
Menolipsis (days)	58.94 ± 5.21	60.07 ± 4.91	0.371

BMI, Body mass index; URM, Unexplained recurrent miscarriage.

## Abbreviations

CCK-8: cell counting kit-8
Co-IP: Co-immunoprecipitation
DAPI: 4',6-diamidino-2-phenylindole
FBS: Fetal bovine serum
GD: Gestational day
IP: Immunoprecipitation
LC-MS/MS: Liquid Chromatography with tandem mass spectrometry
MANF: Mesencephalic astrocyte-derived neurotrophic factor
MDM2: Mouse double minute 2
MMP: Matrix metallopeptidase
NPM1: Nucleophosmin 1
OD: Optical density
PBS: Phosphate buffered saline
PCNA: Proliferating cell nuclear antigen
PMSF: Phenylmethanesulfonyl fluoride
PPI: Protein-protein interaction
PVDF: Polyvinylidene difluoride
RM: Recurrent miscarriage
SDS: Sodium dodecyl sulfate
siRNA: Small interfering RNA
URM: Unexplained recurrent miscarriage
