# Supplementary material for: MANF Promotes Unexplained Recurrent Miscarriages by Interacting with NPM1 and Downregulating Trophoblast Cell Migration and Invasion
Source: Int J Biol Sci. 2024 Jan 1;20(1):296–311. doi: 10.7150/ijbs.85378 (PMC10750294; doi:10.7150/ijbs.85378)
Supplement: Supplementary file 1 — Supplementary figures and table. [file ijbsv20p0296s1.pdf]

Supplementary information:

Figure S1

Schematic representation of the mouse URM model acquisition and experimental conditions.

Figure S2

Screening of HTR-8/SVneo cell lines with different knockdown effects of MANF and NPM1.

A and B. Comparison of mRNA and protein levels of three knockdown MANF lentiviral sequences.

C and D. Comparison of mRNA and protein levels of three NPM1 siRNAs.

Figure S3

NPM1 peptides identified by mass spectrometry in immunoprecipitated URM villi lysates using an anti-MANF antibody. A and B. Primary ion flow diagram of LC-MS/MS method. C, D, E and F. Secondary mass spectra of several high scoring specific peptides of NPM1 identified by LC-MS/MS.

Figure S1

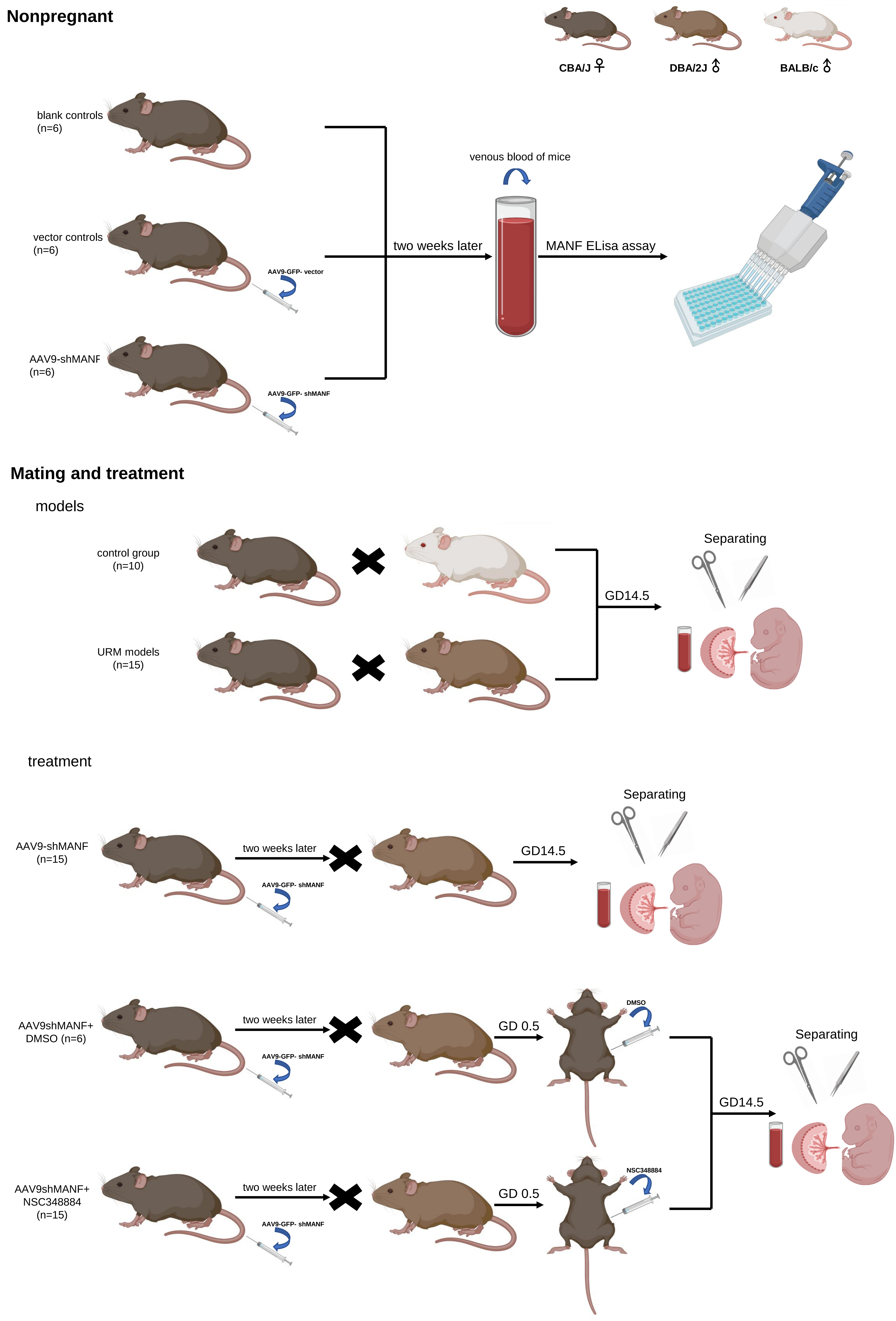

Nonpregnant
CBA/J ♀
DBA/2J ♂
BALB/c ♂
blank controls
(n=6)
vector controls
(n=6)
AAV9-GFP- vector
AAV9-shMANF
(n=6)
AAV9-GFP- shMANF
two weeks later
venous blood of mice
MANF ELisa assay
Mating and treatment
models
control group
(n=10)
URM models
(n=15)
GD14.5
Separating
treatment
AAV9-shMANF
(n=15)
two weeks later
AAV9-GFP- shMANF
GD14.5
Separating
AAV9shMANF+
DMSO (n=6)
two weeks later
AAV9-GFP- shMANF
GD 0.5
DMSO
AAV9shMANF+
NSC348884
(n=15)
two weeks later
AAV9-GFP- shMANF
GD 0.5
NSC348884
GD14.5
Separating

Figure S2

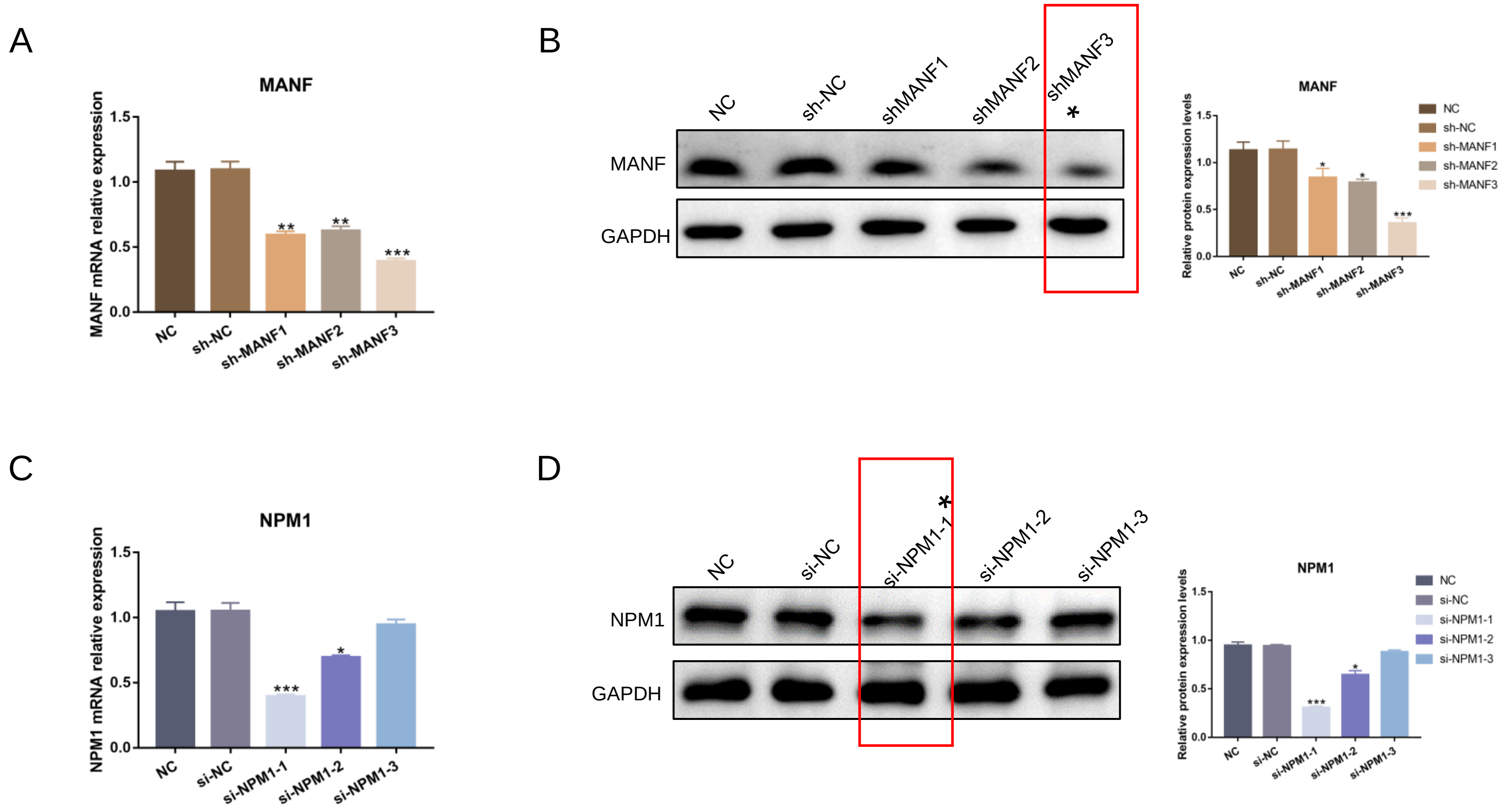
A
MANF
MANF mRNA relative expression
NC
sh-NC
sh-MANF1
sh-MANF2
sh-MANF3
B
NC
sh-NC
shMANF1
shMANF2
shMANF3
MANF
GAPDH
Relative protein expression levels
C
NPM1
NPM1 mRNA relative expression
NC
si-NC
si-NPM1-1
si-NPM1-2
si-NPM1-3
D
NPM1
GAPDH
Relative protein expression levels

Figure S3

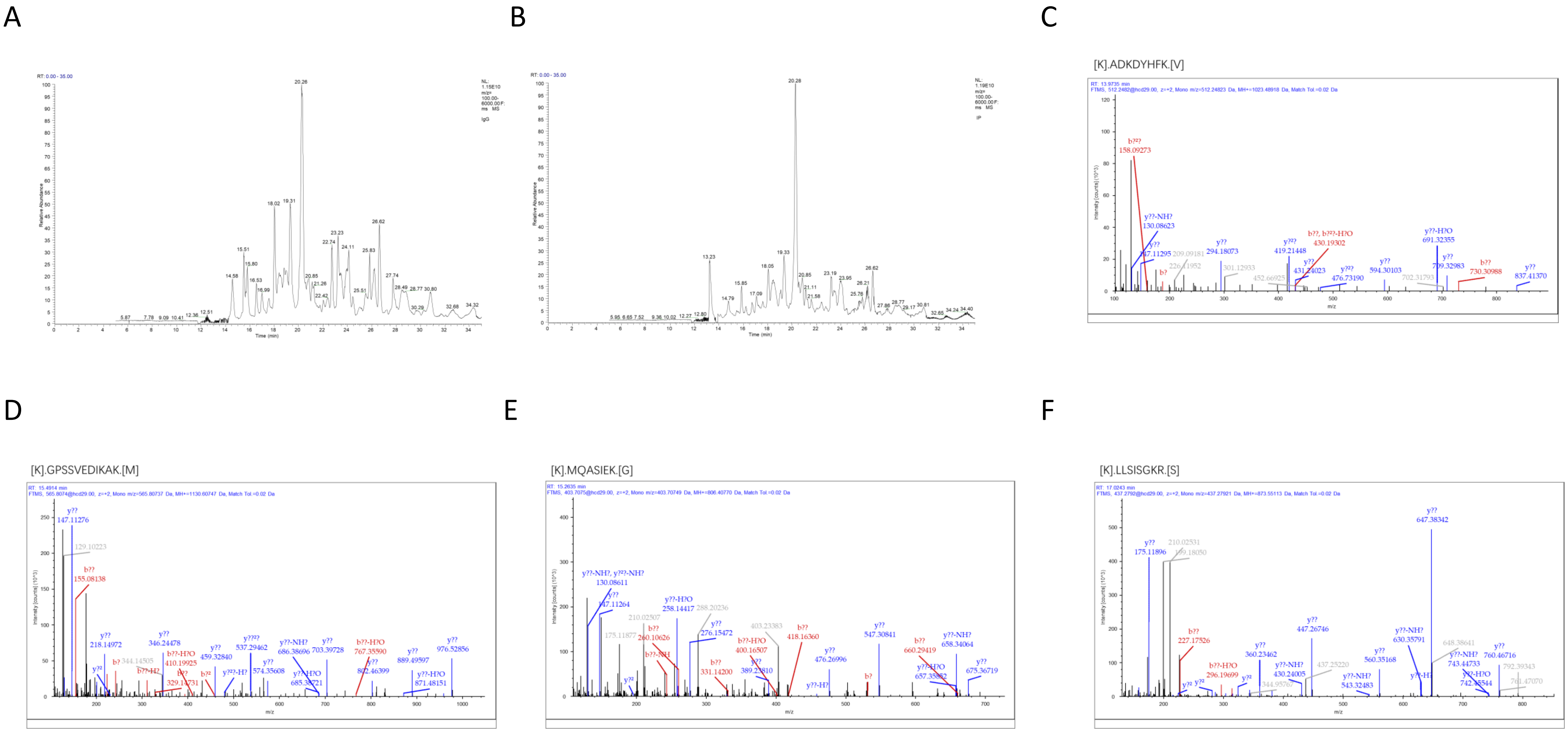

Table S1. Sequence of primers used to amplify target genes

Primer	Primer sequence (5′-3′)
MANF	F: TTTACCAGGACCTCAAAGACAGA
	R: TTGCTTCCCGGCAGAACTTTA
MMP2	F: GATACCCTTGACGGTAAGGA
	R: CCTTCTCCCAAGGTCCATAGC
MMP9	F: TGTACCGCTATGGTTACACTCG
	R: GGCAGGGACAGTTGCTTCT
PCNA	F: GCGTGAACCTCACCAGTATGT
	R: TCTTCGGCCCTTAGTGTAATGAT
NPM1	F: GGAGGTGGTAGCAAGGTTCC
	R: TTCACTGGCGCTTTTTCTTCA
β-ACTIN	F: CAAGATCATTGCTCCTCCTG
	R: ATCCACATCTGCTGGAAGG
